# Is There a Future for PPARs in the Treatment of Neuropsychiatric Disorders?

**DOI:** 10.3390/molecules25051062

**Published:** 2020-02-27

**Authors:** Michele Tufano, Graziano Pinna

**Affiliations:** The Psychiatric Institute, Department of Psychiatry, College of Medicine, University of Illinois at Chicago, 1601 W. Taylor Str., Chicago, IL 60612, USA; mtufan@uic.edu

**Keywords:** PPAR-α, PPAR-γ, neuropsychiatric disorders, major depression, Alzheimer’s disease, allopregnanolone, BDNF, neuroinflammation, toll-like receptor

## Abstract

Recently, peroxisome proliferator-activated receptor (PPAR)-α and γ isoforms have been gaining consistent interest in neuropathology and treatment of neuropsychiatric disorders. Several studies have provided evidence that either the receptor expression or the levels of their endogenously-produced modulators are downregulated in several neurological and psychiatric disorders and in their respective animal models. Remarkably, administration of these endogenous or synthetic ligands improves mood and cognition, suggesting that PPARs may offer a significant pharmacological target to improve several neuropathologies. Furthermore, various neurological and psychiatric disorders reflect sustained levels of systemic inflammation. Hence, the strategy of targeting PPARs for their anti-inflammatory role to improve these disorders is attracting attention. Traditionally, classical antidepressants fail to be effective, specifically in patients with inflammation. Non-steroidal anti-inflammatory drugs exert potent antidepressant effects by acting along with PPARs, thereby strongly substantiating the involvement of these receptors in the mechanisms that lead to development of several neuropathologies. We reviewed running findings in support of a role for PPARs in the treatment of neurological diseases, including Alzheimer’s disease or psychiatric disorders, such as major depression. We discuss the opportunity of targeting PPARs as a future pharmacological approach to decrease neuropsychiatric symptoms at the same time that PPAR ligands resolve neuroinflammatory processes.

## 1. Introduction

**Figure 1:**
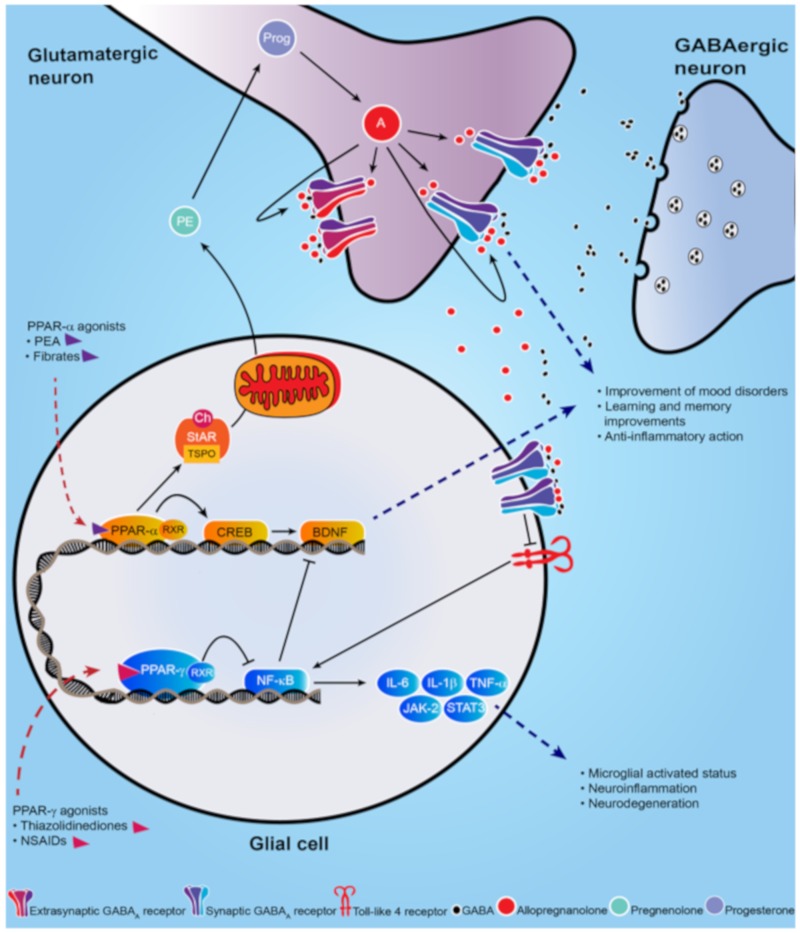
Schematic representation of PPAR-α and PPAR-γ signal cascade following their activation by endogenous or synthetic ligands. PPAR-α endogenous and synthetic agonists, including PEA and the fibrates, activate PPAR-α that dimerizes with the retinoid X-receptor (RXR) and activates the calcium influx through transcriptional regulation of cyclic AMP response element-binding protein (CREB), which in turn promotes hippocampal brain derived neurotropic factor (BDNF) signaling cascade. PPAR-α activation also upregulates both the steroidogenic acute regulatory protein (StAR), which forms a complex with cholesterol and translocator protein (TSPO) allowing the entry of cholesterol into the inner mitochondria membrane where cholesterol is transformed into pregnenolone (PE), the precursors of all neurosteroids, through the cholesterol side-chain cleavage enzyme (P450scc). PE, which is translocated to cortical and hippocampus glutamatergic pyramidal neurons is then converted in allopregnanolone. Allopregnanolone enhances γ-aminobutyric acid action at GABA_A_ receptors [9,10] and improves emotional behavior. Allopregnanolone may also exert an important anti-inflammatory action by binding at α2-containing GABA_A_ receptor subtypes located in glial cells, through inhibition of toll-like 4 receptor/NF-κB pathway [11]. PPAR-γ agonists potentiate the PPAR-γ-induced inhibitory action on NF-κB, which is responsible for microglial activated status, neuroinflammation and neurodegeneration. Moreover, NF-κB inhibits the hippocampal BDNF signaling cascade [12,13]. Thus, PPAR-γ agonists exert an anti-inflammatory effect, by decreasing pro-inflammatory cytokines IL-6, IL-1β, TNF-α, as well as the JAK-2/STAT3 pathway, which is involved in immunity processes. Additionally, activation of PPAR-γ plays a neuroprotective action by decreasing the inhibition on BDNF signaling pathway.

Peroxisome proliferator-activated receptors (PPARs) are non-steroid nuclear receptors, which dimerize with the retinoid X receptor (RXR) and bind to PPAR-responsive regulatory elements (PPRE) in the promoter region of target genes (Figure 1) [1]. PPARs are expressed in many cellular types and tissues and exhibit differences in ligand specificity and activation of metabolic pathways [2]. In humans, among all known transcriptional factors belonging to the nuclear receptor superfamily, three isoforms of PPARs have been characterized: PPAR-α, PPAR-β/δ, and PPAR-γ, also known as *NR1C1, NR1C2,* and *NR1C3*, respectively [3]. PPARs are a target for fatty acids (unsaturated, mono-unsaturated, and poly-unsaturated), for which they mediate binding and transport, as well as oligosaccharides, polyphenols, and numerous synthetic ligands [4]. Furthermore, they are involved in a series of molecular processes, ranging from peroxisomal regulation and mitochondrial β-oxidation to thermogenesis and lipoprotein metabolism [5]. PPAR distribution changes in different organs and tissues. In rodent central nervous system, the three isoforms are widely co-expressed across brain areas and in circuitry that are responsible for mediating stress-responses, which supports a role in several neuropsychopathologies by mediating anti-inflammatory and metabolic actions [6,7]. Intriguingly, both synthetic and endogenously-produced PPAR agonists have shown benefits for treatment of mood disorders and neurological diseases [8].

**Figure 2:**
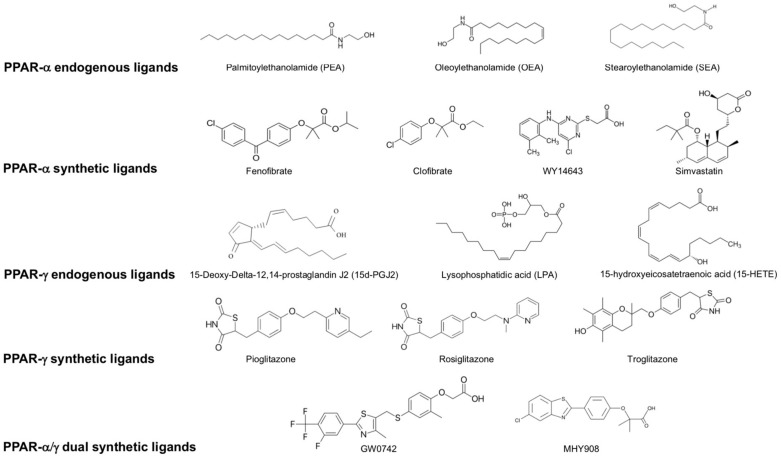
List of endogenous and synthetic PPAR- α, PPAR-γ and dual PPAR- α/γ ligands.

By enhancing free fatty acid uptake, PPARs may improve insulin sensitivity and beta-cell properties in hyperglycemia in patients affected with type 2 diabetes [14]. For example, thiazolidinediones, including pioglitazone and rosiglitazone, are synthetic ligands that selectively bind at PPAR-γ and are used clinically for the treatment of diabetes [15]. However, given their side effects on weight gain, congestive heart failure, bone fractures, and macular and peripheral edema, the Food and Drug Administration (FDA) has limited their use [16]. PPAR-α synthetic ligands, including the fibrates (fenofibrate, clofibrate) (depicted in Figure 2) are characterized by a much safer pharmacological profile and are widely prescribed to lower high cholesterol blood levels and triglycerides [17]. While PPAR-α and γ endogenous and synthetic ligands have been well characterized for the treatment of diabetes and cardiovascular disease, their central neuronal effects on behavior and neuropathology have only emerged recently [7].

The efficacy of PPAR-γ agonists on behavior was initially shown in rodent models of anxiety and depression, where the administration of rosiglitazone significantly reduced the immobility time in the forced swim test [18]. This antidepressant effect was also observed in clinical trials where administration of pioglitazone or rosiglitazone improved symptoms in patients with major depression [16]. Importantly, the improvement in depression correlated with normalization of inflammatory biomarkers (e.g., IL-6) and insulin resistance, suggesting an intriguing link among PPAR-γ-activation, depression, inflammation, and metabolism [16].

These findings highlight the potential therapeutic value of PPAR-γ agonists in the treatment of neuropsychiatric disorders [16,18,19]. Furthermore, they encourage developing new antidepressant drugs beyond the traditional selective serotonin reuptake inhibitors (SSRIs). SSRIs are relatively inefficient because they only improve symptoms in about half of patients with mood disorders, including major depression and post-traumatic stress disorder (PTSD) [9]. Hence, there is an urgent need for developing new treatment strategies and identifying novel neurobiological targets and biomarkers that may stimulate discovery of novel ligands [9].

Recently, neuroinflammation has been the focus of new scientific evidence that is convincingly demonstrating its contribution to neuropathophysiology of mood disorders [20]. Several studies have recently observed elevated peripheral and central neuroinflammatory markers in psychiatric disorders, such as PTSD, suicidal behavior, and schizophrenia [21,22,23]. It is remarkable that patients with high levels of neuroinflammation respond poorly to classical antidepressants, suggesting that targeting neuroinflammatory pathways may offer a therapeutic strategy to revert or alleviate mood symptoms as well [24]. Intriguingly, dietary interventions have been tested in several neuropsychiatric disorders, such as multiple sclerosis (MS), anxiety, and depression [25,26]. As molecular targets for various natural ligands found in a number of aliments, PPARs may shed light into the molecular mechanisms underlying the success of dietary treatments in nutritional psychiatry [27].

Herein, we review recent findings on the emerging role of PPAR-α and PPAR-γ in the treatment of neuropsychiatric disorders with a particular focus on their role in the regulation of inflammation.

## 2. Brain Distribution of PPARs in the Rodent Brain

The distribution of PPARs changes over different tissues. In rat brain, the three isoforms are co-expressed during neurodevelopment. Later in life, PPAR-β/δ becomes the most predominant isoform and subsequently there is a decrease in the expression of PPAR-α and PPAR-γ [28]. However, PPAR-α is widely expressed in amygdala, prefrontal cortex, thalamic nuclei, nucleus accumbens, ventral tegmental area, and basal ganglia [29,30]. In these regions, PPAR-α expression is found at the highest levels in neurons, followed by astrocytes, where it was detected in cell body and astrocytic processes, and is weakly expressed in microglia. PPAR-α is the only isotype that colocalizes with all cell types and it is the most expressed isotype in astrocytes [30]. PPAR-β/δ has a ubiquitous distribution across the brain. It is the most widely expressed isotype both in the brain and the periphery, and studies suggest a regulatory role for PPAR-β/δ on the other isoforms [31]. PPAR-γ is highly expressed in the amygdala, piriform cortex, dental gyrus and basal ganglia, with lower levels in thalamic nuclei and hippocampal formation [6,30]. PPAR-γ is also more widely expressed in neurons than astrocytes where it varies across brain regions, with higher expression in nucleus accumbens followed by the prefrontal cortex. Interestingly, PPAR-γ is not expressed in microglia of mouse or human brain unless there is a condition of a microglial functional state, as it appears after lipopolysaccharide (LPS) treatment [30]. While PPAR-β/δ is not found in microglia, PPAR-α is the only isotype expressed under normal and LPS conditions [30]. PPAR expression across brain areas in circuitry that regulates stress-response suggests that they may play a role in several neuropsychopathologies by mediating anti-inflammatory and metabolic actions. Intriguingly, both synthetic and endogenously-produced PPAR agonists have shown benefits for treatment of mood disorders and neurological diseases [7,8,10,32,33].

## 3. Neuropsychiatric Disorders and PPARs

### 3.1. Mood Disorders

Major depressive disorder (MDD) affects 15%–20% of the general American population, thereby representing a remarkable burden for society, exacerbated by its chronicity and comorbidity with other prevalent mood disorders, such as PTSD and suicide, and drug use disorder [34,35]. It is the second most common cause of disability worldwide and it is expected to become the main cause in high-income countries by 2030 [36]. Currently, FDA-approved treatments for depression include the SSRI antidepressants and the serotonin-norepinephrine reuptake inhibitor (SNRIs), which have a high rate of non-responders [37]. Additionally, these medications might take up to several weeks to induce pharmacological effects and patients often drop-off treatment because of a variety of unwanted secondary effects, which comprise insomnia, headache, sexual dysfunction, and dry mouth [38]. While novel therapeutic strategies are urgently needed for the management of MDD, PTSD, and other mood disorders, the nuclear receptors PPAR-α and γ are gaining consistent interest as new promising targets for treating behavioral dysfunction (please see Table 1 and Table 2 for a summary) [39]. This is further substantiated by the recent discovery that stimulation of PPAR-α can enhance neurosteroid biosynthesis [10], which is implicated in the etiopathology of mood disorders and their treatment [7,40,41,42,43].

#### 3.1.1. PPAR-α

PPAR-α selective agonists (Figure 2) have been associated with antidepressant effects in murine models of stress-induced depression [44,73]. The PPAR-α agonist WY14643 improved depressive-like behavior in the chronic social defeat stress model, in the tail suspension test, and in forced swim test, commonly used rodent paradigms to evaluate depression-like behavior [74]. The anti-depressant activity of WY14643 appeared to involve activation of the brain derived neurotropic factor (BDNF) signaling cascade [44], a pathway implicated in a range of neuronal processes (depicted in Figure 1) [75,76,77,78]. It is well known that BDNF levels are found to be decreased in the hippocampus of depressed individuals and in animal models of this disorder [79,80,81,82]. Furthermore, the action of antidepressants, like fluoxetine, increase BDNF levels, which in turn correlates with an improvement of behavioral dysfunction [83]. Fenofibrate, another selective synthetic agonist of PPAR-α (depicted in Figure 2), is a fibric acid derivative largely prescribed in the treatment of primary hypercholesterolemia [84]. Similar to WY14643, fenofibrate administration in a rodent model of depression exerts antidepressant-like effects via activation of PPAR-α-mediated promotion of hippocampal BDNF signaling cascade [45]. Fenofibrate also improved anxiety-like behavior in an animal model of PTSD [10].

PPAR-α is also involved in hippocampal long-term memory processes by upregulating plasticity-related genes [85]. Intriguingly, PPAR-α is widely expressed in hippocampal neurons, where it controls the calcium influx through transcriptional regulation of cyclic AMP response element binding (CREB) [86,87]. The PPAR-mediated transcriptional regulation of CREB also stimulates BDNF expression, which improves learning and memory in animal models of Alzheimer’s disease [88]. Interestingly, the HMG-CoA reductase inhibitor, simvastatin, which is used to decrease triglycerides levels and reduce the risk of heart disease, is also able to reverse the depression-like behaviors induced in rats by chronic mild stress (CMS) [46]. This action also appears to be mediated by enhancing the hippocampal expression of BDNF signaling pathway via the PPAR-α-mediated activation of CREB [88]. Consistent with these findings, in a chronic stress-induced mouse model of depression, PPAR-α expression is decreased in the hippocampus, which in turn results in reduced hippocampal BDNF expression [89]. Conversely, genetic overexpression of PPAR-α induces antidepressant effects by a CREB-mediated biosynthesis of BDNF. Both genetic or pharmacological inhibition of PPAR-α blocks the anti-depressive effects of fluoxetine, thereby suggesting its involvement in the molecular mechanisms of antidepressant drug action [89].

Downregulation of PPAR-α expression was recently associated with the molecular mechanisms underlying behavioral deficits [10]. Likewise, its anti-depressive pharmacological effects correlated with the stimulation of neurosteroid biosynthesis [10]. The main PPAR-α endogenous agonist, N-palmitoylethanolamine (PEA) is an anti-inflammatory, analgesic, and anti-allergic compound clinically tested for its neuroprotective effects in multiple sclerosis (MS), Alzheimer’s disease (AD), and Parkinson’s disease (PD) [90,91]. PEA can be produced endogenously or acquired through plant-based food sources and it is endogenously metabolized by the fatty acid amide hydrolase (FAAH) [91], which is an enzyme involved in the metabolism of endocannabinoids, including anandamide (AEA) [92]. As an endogenous ligand, PEA activates the G-protein coupled receptor, GPR55, while showing low affinity for the cannabinoid receptor type-1 (CB1) and type2 (CB2) [93]. However, its therapeutic behavioral effects appear to be mediated via PPAR-α binding and activation [10,94]. PEA administration in socially isolated mice, a model of protracted stress-induced PTSD [48,49], normalized reduced brain levels of allopregnanolone, a GABAergic neurosteroid, which is found decreased in patients with depression and PTSD [95,96,97,98,99]. In the socially isolated mouse, PEA improved contextual fear responses and facilitated contextual fear extinction and fear extinction retention, as well as ameliorated depressive-like and anxiety-like behavior by increasing corticolimbic levels of allopregnanolone [10]. Consistently, in a cohort of Ugandan war survivors affected by PTSD, the hair levels of PEA, oleoylethanolamide (OEA), and stearoylethanolamide (SEA) were found to be decreased when compared with levels of war survivors without current or lifetime PTSD [100], thus suggesting a decreased PPAR-α signal pathway in PTSD. While it is important that these findings will be confirmed also in blood and post-mortem brain of PTSD patients [101], this observation provides support to the involvement of the PPAR-allopregnanolone axis dysfunction in PTSD. Together with the findings that allopregnanolone has been found decreased in cerebrospinal fluid (CSF) and plasma of both male and female PTSD and MDD patients [95,97,98,99,102], these clinical data provide a translational example with PTSD animal models [103].

#### 3.1.2. PPAR-γ

In 2009, a case report showed that pioglitazone improved symptoms in a 55-year-old woman affected by unipolar major depression [104]. In another study, pioglitazone (Figure 2) was administered in a 24-week double-blind randomized clinical trial in 145 patients with a metabolic syndrome [65]. Patients showed improvement in anxiety and depression symptoms following pioglitazone treatment. Furthermore, pioglitazone showed anxiolytic effects in nondiabetic insulin-resistant patients [65]. In a double-blind placebo-controlled clinical trial with patients not showing any metabolic syndrome or diabetes, pioglitazone was beneficial in the treatment of MDD [105]. Administration with pioglitazone (15–30 mg/day) given for 8 weeks to a cohort of 34 patients with bipolar depression also improved depressive symptoms [66]. However, when tested for efficacy in a 8-week, double-blind, randomized, placebo-controlled trial of 37 patients, pioglitazone (15–45 mg/day) failed to improve bipolar depression symptoms [67].

In preclinical studies, administration with pioglitazone improved depression-like behavior induced by LPS [12]. After pioglitazone administration, the NF-κB/IL-6/STAT3 pathway was inhibited with concomitant down-regulation of the CREB/BDNF pathway [12,13]. In a mouse model of depression, the pharmacological effects of pioglitazone in improving behavioral deficits involved central serotonergic neurotransmission [12]. In mice exposed to CMS, administration of pioglitazone (2.5 mg/kg) decreased the CMS-induced microglial activated status (Iba1 +) in the hippocampus and improved the microglial neuroprotective phenotype, resulting in an overall amelioration of depressive-like behavior [47]. Interestingly, this behavioral improvement was associated with the inhibition of microglia-mediated neuroinflammation [47]. Indeed, after pioglitazone treatment, the expression of pro-inflammatory molecules (IL-1β, IL-6, and TNFα) was reduced and the levels of anti-inflammatory cytokines (IL-4, IL-10, and TGF-β) were increased in CMS mice [47]. Moreover, decreased PPAR-γ mRNA and protein expression in adipose tissue were correlated with chronic social defeat stress. Rosiglitazone also elicited antidepressant and anxiolytic-like effects through the PPAR-γ-mediated decrease in adiponectin production, suggesting that the PPAR-γ-adiponectin axis may be involved both in metabolism and stress-related homeostasis [106].

### 3.2. Neurodevelopmental Disorders

The involvement of PPARs in psychiatric disorders is not limited to MDD and PTSD. Schizophrenia has been associated with increased inflammation, suggesting that the neuroinflammatory processes may play a role in the pathophysiology of this prevalent mental disorder [107]. In peripheral blood mononuclear cells (PBMC) extracted from chronic schizophrenic patients, a decreased expression and activity of PPAR-γ correlated with lower plasma levels of its endogenous ligand, 15d-prostaglandin J2, which overall indicates a state of increased inflammation [108]. Another study in patients affected by schizophrenia investigated the expression of inflammatory and metabolic genes [109]. Expression of PPAR-γ was increased while PPAR-α was decreased, suggesting a metabolic-inflammatory imbalance in schizophrenia [109]. Pioglitazone provided benefits in reversing this metabolic condition. Also, administration of pioglitazone to the GluN1 knockdown model of schizophrenia improved long-term memory and helped to restore cognitive endophenotypes [50]. Unlike most anti-psychotics, pioglitazone had a restorative effect on cognitive function (measured by the puzzle box assay), thus suggesting a potential impact of pioglitazone as an augmentation therapy in schizophrenia [50,110].

Additionally, for its anti-inflammatory and neuroprotective properties [111], PPAR-α activation by fenofibrate prevented prenatal maternal immune activation, which in a rat model is associated with the risk of developing schizophrenia in the offspring [112]. Remarkably, prenatal administration of pioglitazone dampens the schizophrenia-like behavior observed in male offspring after prenatal maternal immune activation [112].

Autism spectrum disorder (ASD) is also characterized by neuroinflammation, oxidative stress and depletion of glutathione in the brain [113]. In clinical studies, pioglitazone was tested in a 16-week prospective cohort of 25 autistic children, showing good tolerability and leading to a statistically significant improvement in repetitive behaviors, social withdrawal, and externalizing behaviors [70]. However, randomized controlled trials are still needed in order to fully validate pioglitazone as valuable treatment for ASD [70].

In preclinical studies, administering pioglitazone decreases inflammation and oxidative stress, thus reverting the ASD-like behavior [114]. The ASD-like behavioral deficits can be induced in rats with a postnatal treatment with propionic acid, which increases inflammation and oxidative stress [51]. Treatment with pioglitazone from postnatal day 24, mitigated the ASD-like behavior and reduced oxidative stress and inflammation by reducing IL-6 and TNF-α while increasing IL-10 in cerebellum, prefrontal cortex and brainstem [115]. A natural ligand of PPAR-γ is resveratrol, which is also able to prevent social behavioral impairments in a rodent ASD model [116,117].

Consistent with a PPAR-α activation, neurobehavioral and biochemical benefits in an ASD animal model were observed following administration with fenofibrate that resulted in reduced oxidative stress and inflammation in several brain regions [52]. PPAR-α is required for normal cerebral functions and its genetic ablation leads to repetitive behaviors and cognitive inflexibility in mice [118]. In another rodent model of ASD, the BTBR T + tf/J (BTBR) mouse, PEA reverted the altered phenotype and improved ASD-like behavior through a PPAR-α activation. This effect was accompanied by decreased levels of inflammatory cytokines in serum, hippocampus, and colon [53]. PEA administration restored the hippocampal BDNF signaling pathway in BTBR mice and improved mitochondrial dysfunction, which has been observed in ASD (Table 1 and Table 2) [53].

PPAR-β/δ has also an effect in improving inflammation related to ASD [119]. Treatment with the selective agonist, GW0742, improved repetitive behaviors and lowered thermal sensitivity responses in the BTBR rodents, while decreasing pro-inflammatory and increasing anti-inflammatory cytokines [54].

These findings strongly support the involvement of PPARs in the neuropathology of mood and neurodevelopmental disorders [100]. A PPAR-α-allopregnanolone (i.e., endocannabinoid-like/neurosteroids) cross-talk may have an impact for establishing relevant novel targets for the treatment of PTSD and major depression [9]. Intriguingly, these newly observed link between the endocannabinoid-like system and biosynthesis of neurosteroids may additionally provide bio-signatures for the diagnosis and treatment of psychiatric disorders, which still rely on subjective measures based on the DSM-5 criteria [120]. Furthermore, PPAR-γ agonists, including pioglitazone, have shown promising antidepressant effects in several clinical trials [16,65,66,67]. It is also remarkable that non-steroidal anti-inflammatory drugs, including ibuprofen and aspirin, whose mechanism of action includes a PPAR-γ activation, have consistently shown potent antidepressant effects [121].

Collectively, these studies involving PPAR-α and γ in the neuropathophysiology of psychiatric disorders have opened up new opportunities for the development of new therapeutics, which represent a novel emerging field of neuropsychopharmacology [18,66,89,122].

### 3.3. Neurological Disorders

In Alzheimer’s disease (AD) the deposition of amyloid β triggers neuroinflammation and oxidative damage [122,123]. However, the pathogenic mechanisms for AD onset and progression are far from being elucidated. For their involvement in neuroinflammation, oxidative stress and energy metabolism, PPARs have been considered as promising therapeutic target for AD (please see Table 1 and Table 2) [123,124]. PPAR-γ signaling is coordinated with the Wnt/beta-catenin signaling in opposite ways. Wnt/beta-catenin is downregulated when PPAR-γ is upregulated in AD [125]. Imbalance in the Wnt/beta-catenin/PPAR-γ regulation plays a role in physiopathology of neurological disorders owing to its involvement in oxidative stress and cell death through regulation of metabolic enzymes [125]. Administration of pioglitazone in a genetically modified AD mouse model showed reductions in both soluble and insoluble amyloid β, while improving memory, learning deficits, and preventing neurodegeneration [126]. However, in clinical studies, pioglitazone showed no significant effects on cognitive outcomes [62,71].

To remediate the effects of neurodegeneration, the agonists L165, 041, and F-L-Leu, acting on PPAR-β/δ and PPAR-γ, respectively, have been simultaneously administered in a Streptozotocin rat model of AD [63]. The treatment improved myelin and neuronal maturation, mitochondrial proliferation, and function, while decreasing neuroinflammation, indicating a role, not only for PPAR-γ, but also for PPAR-β/δ in the pathology of AD [63]. On the other hand, activation of PPAR-α by PEA has proven efficacy in inhibiting amylogenesis, neuroinflammation, neurodegeneration and Tau hyperphosphorylation [124]. Whether PEA could play a role alone or adjuvant of other AD therapeutics should be further investigated [127]. The Aβ-induced tau protein hyperphosphorylation is also reduced by cannabidiol (CBD) administration, through the PPAR-γ and Wtn/β-catenin stimulation, which underscores a role for this phytocannabinoid in reducing neuroinflammation and oxidative stress [128].

Analogously, owing to their engagement in the regulation of neuroinflammation and innate immune response, PPAR dysfunction may play a role in the molecular mechanisms that trigger multiple sclerosis (MS) [129,130]. Interestingly, in PBMC of patients affected by MS, PPAR-γ expression was decreased [131, 132]. Troglitazone (a PPAR-γ agonist) administration attenuated inflammation and ameliorated experimental autoimmune encephalomyelitis (EAE), an animal model of MS [64]. Moreover, a polymorphism (Pro12A) on the PPAR-γ gene is correlated with higher risk of delayed MS onset [133]. When tested for efficacy in a cohort of 24 patients, pioglitazone showed no improvement in clinical symptoms after 1 year of treatment, although a decrease in grey matter atrophy and reduced lesion burden was observed using magnetic resonance imaging (MRI) [72].

Parkinson’s disease (PD) symptoms have been reproduced in rodent models by administering 1-methyl-4-phenyl-1,2,3,6-tetrahydropyridin (MPTP), which induces degeneration of dopaminergic neurons in the substantia nigra [134]. When administered in this model, pioglitazone protects against MPTP-induced neurotoxicity, decreasing microglial activation, and iNOS-positive cells, as well as inhibiting monoamine oxidase-B expression. In a chronic model of MPTP, rosiglitazone administration protected from loss of dopaminergic neurons and prevented olfactory and motor alteration [55,56,57]. MHY908, a PPAR-α/γ dual agonist, exerts neuroprotective effects by reducing microglial activation and neuroinflammation, thereby diminishing dopaminergic neuronal damage in a MPTP mouse model of PD [135]. Similarly, the PPAR-γ agonist, MDG548 mediates neuroprotection in LPS-stimulated microglia and, in the MPTP mouse model, by boosting phagocytosis and anti-inflammatory cytokines production (e.g., IL-10) [136]. This further supports PPAR-γ engagement in microglial function, phagocytosis and neuroinflammation. The overexpression of PPAR-γ coactivator-1α (PGC-1α), a master regulator of mitochondrial metabolism and oxidative stress [137], protects MPTP-induced dopaminergic neuronal degeneration. Interestingly, resveratrol-mediated PGC-1α activation also protected against dopaminergic neuronal degeneration in the MPTP mouse model, with efficiency comparable to the PGC-1α overexpression [138]. This finding suggests that resveratrol and other compounds, which act on PPAR-γ and PGC-1α might be beneficial as therapeutic agents in PD pathophysiology and possibly in other neurological disorders [138].

PPARs have also shown beneficial effects in improving epilepsy, a chronic disorder characterized by unprovoked seizures, which affects 1% of the human population [139]. Administration of a long-term fenofibrate diet negatively modulated the nicotine-induced increase of large inhibitory postsynaptic currents recorded in pyramidal neurons and improved motor-behavioral seizures [140]. Therefore, PPAR-α has been proposed as a therapeutic target for nocturnal frontal lobe epilepsy, which is a form of idiopathic epilepsy with an autosomal inherited component [140]. PPAR-α also plays a role in the duration and occurrence of seizures (measured by a spike-wave discharges on EEG recordings) in WAG/Rij rats, one of the most used models of human absence epilepsy [141], where PEA attenuates seizures by binding PPAR-α and indirectly by activating the CB1 receptor [61].

Altogether, the results obtained from testing PPAR-α and γ agonists (such as fenofibrate, pioglitazone, resveratrol and rosiglitazone) in various neurological disorders support PPAR-α and PPAR-γ as potential novel targets for the therapeutic management of prevalent and debilitating conditions, such as Alzheimer’s and Parkinson’s disease. Undoubtedly, more clinical trials are required to demonstrate the efficacy and safety of PPAR agonists and, considering the side effects linked to treatment with some PPAR-γ agonists, it would be necessary to explore alternative strategies for administration, as well as adjusting for doses and delivery of drugs. Further understanding of the molecular mechanism of PPAR’s role in neuroinflammation, which is emerging as a common process in neuropsychiatric disorders, as well as, a better comprehension of their activation by endogenous ligands, which can also be introduced by food sources, is also needed.

## 4. Conclusions

PPARs are implicated in a variety of molecular processes that bridge metabolism to inflammation, where they have been extensively studied. PPAR-α and PPAR-γ synthetic ligands (e.g., fenofibrate, rosiglitazone) have been approved by the FDA for treatment of high cholesterol levels and diabetes, respectively. Remarkably, their role in the regulation of behavioral dysfunction is just emerging and their engagement in the pathophysiology and treatment of psychiatric and neurological disorders is becoming an intriguing new treatment opportunity to manage these conditions. In this respect, PPAR’s involvement in neuroinflammatory processes associated with psychiatric and neurological disorders is relevant not only for discovering novel therapeutics that activate PPARs, but also for exploring new biomarker candidates that may help with the prevention and diagnosis of these debilitating conditions [9,33]. Furthermore, the finding that endogenous ligands, including PEA or other natural ligands are found in aliments, opens the field of nutritional psychiatry to investigate micronutrients that activate PPARs [142]. This trend may lead to developing natural therapeutic approaches for treating neuropsychiatric disorders through functional foods.

## 5. Patents

Graziano Pinna has a patent pending application on PEA and PPAR-α agonists in the treatment of neuropsychiatric disorders.

## Figures and Tables

**Table 1:** Studies of peroxisome proliferator-activated receptor (PPAR) ligands in models of neuropsychiatric disorders.

Preclinical Studies					
**Models of mood disorders**					
**Disease**	**Model**	**Agonist**	**Molecular Target**	**Effect**	**References**
**Depression**	GW-9662 treatment	Rosiglitazone	PPAR-γ	Antidepressant effect; reduce the immobility time in the forced swim test	[18]
	Chronic social defeat stress	WY14643	PPAR-α	Improve depressive-like behavior in the tail suspension test and forced swim test	[44]
	Chronic social defeat stress	Fenofibrate	PPAR-α	Antidepressant-like effects	[45]
	CMS-exposed rats	Simvastatin	PPAR-α	Reverse the depression-like behaviors promoting BDNF signaling pathway	[46]
	CMS-exposed mice	Pioglitazone	PPAR-γ	Decrease microglial activated status (Iba1+) and pro-inflammatory cytokines	[47]
**PTSD**	Socially isolated mice	Fenofibrate PEA	PPAR-α	Increase brain levels of allopregnanolone; Improve anxiety-like behavior; facilitate contextual fear extinction and fear extinction retention	[10], [48], [49]
**Models of neurodevelopmental disorders**					
**Schizophrenia**	GluN1 knockdown	Pioglitazone	PPAR-γ	Improve long-term memory and help restoring cognitive endophenotypes	[50]
**ASD**	Propionic acid autism-like rat	Pioglitazone (from postnatal day 24)	PPAR-γ	Mitigate the ASD-like behavior and reduce oxidative stress and inflammation	[51]
	VPA-autism like Wistar rat	Fenofibrate	PPAR-α	Reduce oxidative stress and inflammation in several brain regions	[52]
	BTBR	PEA	PPAR-α	Revert the altered phenotype and improve ASD-like behavior	[53]
	BTBR	GW0742	PPAR-β/δ	Improve repetitive behaviors and lowers thermal sensitivity responses; decrease pro-inflammatory cytokines	[54]
**Model of neurological disorders**					
**PD**	MPTP	Pioglitazone	PPAR-γ	Protect against neurotoxicity; decrease microglial activation and iNOS-positive cells	[55]
	MPTP	Rosiglitazone	PPAR-γ	Protect from dopaminergic neurons loss; prevents olfactory and motor alteration	[56], [57]
	MPTP	MHY908	PPAR-α/γ dual agonist	Neuroprotective effects; reduce microglial activation and neuroinflammation	[58]
	MPTP	MDG548	PPAR-γ	Mediate neuroprotection in microglia; promote anti-inflammatory cytokines	[59]
	MPTP	Pioglitazone	PPAR-γ	Decrease microglial activation and iNOS-positive cells	[60]
**Epilepsy**	WAG/Rij rats	PEA	PPAR-α	Attenuate seizures	[61]
**AD**	Genetically modified AD mouse	Pioglitazone	PPAR-γ	Improve memory and learning deficits; prevent neurodegeneration	[62]
	Streptozotocin rat L165, 041 and F-L-Leu	L165, 041 and F-L-Leu, simultaneously	PPAR-β/δ and PPAR-γ,	Improve myelin and neuronal maturation, mitochondrial proliferation and function; decrease neuroinflammation	[63]
**MS**	EAE	Troglitazone	PPAR-γ	Attenuate inflammation	[64]

**Table 2:** Effects of PPAR ligands in neuropsychiatric disorders.

Clinical Studies					
**Mood disorders**					
**MDD**	Clinical Trial	Rosiglitazone	PPAR-γ	Improve symptoms; normalize pro-inflammatory cytokines	[16]
	Double-blind, randomized clinical trial; 24-week.	Pioglitazone	PPAR-γ	Improve anxiety and depression	[65]
	Bipolar depression	Pioglitazone (15–30 mg/day for 8 weeks)	PPAR-γ	Improve depressive symptoms	[66]
	Double-blind, randomized, placebo-controlled trial	Pioglitazone (15–45 mg/day for 8 weeks)	PPAR-γ	Fail to improve bipolar depression symptoms	[67]
	Double-blind, randomized, placebo-controlled trial	Pioglitazone (30 mg/day for 12 weeks)	PPAR-γ	Differential improvement according to metabolic and depressive status	[68]
	Double-blind, randomized, placebo-controlled trial	Palmitoylethanolamide (PEA)	PPAR-α	Improve depressive symptoms	[69]
**Neurodevelopmental disorders**					
**ASD**	16-week prospective study of autistic children	Pioglitazone	PPAR-γ	Improve repetitive and externalizing behaviors, social withdrawal	[70]
**Neurological disorders**					
			
**AD**	Double-blind, randomized, placebo-controlled trial	Pioglitazone (45 mg/day for 18 months)	PPAR-γ	No significant effect	[71]
**MS**	Clinical trial, 12 month-treatment	Pioglitazone	PPAR-γ	No improvement in clinical symptoms; decrease grey matter atrophy	[72]
